# Supplementary material for: PARC: Physics-aware recurrent convolutional neural networks to assimilate meso scale reactive mechanics of energetic materials
Source: Sci Adv. 2023 Apr 28;9(17):eadd6868. doi: 10.1126/sciadv.add6868 (PMC10146890; doi:10.1126/sciadv.add6868)
Supplement: Supplementary file 1 — Supplementary Text Figs. S1 to S15 Legends for movies S1 to S10 References [file sciadv.add6868_sm.pdf]

Science Advances
AAAS

Supplementary Materials for

PARC: Physics-aware recurrent convolutional neural networks to assimilate meso scale reactive mechanics of energetic materials

Phong C. H. Nguyen *et al.*

Corresponding author: H. S. Udaykumar, hs-kumar@uiowa.edu; Stephen S. Baek, baek@virginia.edu

Sci. Adv. **9**, eadd6868 (2023)
DOI: 10.1126/sciadv.add6868

The PDF file includes:

Supplementary Text
Figs. S1 to S15
Legends for movies S1 to S10
References

Other Supplementary Material for this manuscript includes the following:

Movies S1 to S10

Supplementary Materials

Additional Details on the PARC Architecture Design

U-Net for Shape Descriptor Extraction The U-Net is an encoder-decoder-based neural network that is widely used for semantic segmentation tasks (36). As illustrated in Fig. S.1A, the U-Net employed in this work is comprised of two network paths: encoder (compression) and decoder (expansion). The encoder takes a 240×240 $(spatial) \times 1$ grayscale microstructure image $I(x, y)$ and a $240 \times 240 \times 1$ position map $U(x, y)$ as inputs. First, two convolution layers with the rectified linear unit (ReLU) activation produce $240 \times 240 \times 64$ low-level feature maps. Consequently, the low-level feature maps are then down-sampled using the 2×2 max-pooling operation with the stride of two, in order to reduce the spatial dimensions of the feature maps by half and thereby double the size of the receptive field. The down-sampled feature maps are processed through two subsequent convolutional layers, resulting in $120 \times 120 \times 128$ feature maps. Kernel sizes are 5×5 for all convolutional layers. A similar process is applied two more times, such that at each time the spatial dimensions of feature maps reduce by half and the number of feature maps increases twice, resulting in throughputs of the size $60 \times 60 \times 256$ and $30 \times 30 \times 512$, respectively. The final $30 \times 30 \times 512$ feature map is a high-level abstraction representation of the original microstructure image. The high-level feature map is then reconstructed by the decoder whose architecture is symmetric to the encoder, to produce a map of shape descriptors that can be used in the derivative network. Unlike the conventional use of U-nets, the U-Net subnetwork in PARC outputs a 128-channel feature map, containing shape descriptors across different image locations. Enabled by the multi-level, multi-resolution architecture of the U-Net, these shape descriptors codify local morphological characteristics of the microstructure in a hierarchical manner.

Differentiator and Integrator CNNs As illustrated in Figs. S.1B and S.1C, the architectures of the differentiator and the integrator are identical, except for the input layers. The differentiator network has $240 \times 240 \times 130$ input dimensions constituted by $\mathbf{X} \in \mathbb{R}^{240\times240\times2}$, the temperature and pressure fields collocated on the simulation grid, and $\mu \in \mathbb{R}^{240\times240\times128}$, the microstructure shape descriptor. The output of the differentiator is the time derivative $\partial\mathbf{X}/\partial t$ of the state $\mathbf{X}$, which then feeds the integrator. Hence the input to the integrator has the dimensions of $240 \times 240 \times 2$. Aside from the input layers, both of the networks are comprised of two ResNet blocks (40) and a super-resolution block (41) of the identical architecture. The first ResNet block contains three 3×3 convolution layers with zero padding to produce a $240 \times 240 \times 64$ feature map. The ResNet skip connection adds the output of the first convolution layer to that of the last convolution layer of the ResNet block. The second ResNet block is the same as the first ResNet block, except for having 128 channels in the output. The super-resolution block starts with a 7×7 convolution layer to produce a $240 \times 240 \times 128$ feature map, followed by two 1×1 convolution layers to produce 240×240 feature maps with the number of channels 64 and 32, respectively. The final output of the SR block is then processed through a 3×3 convolution layer, producing the final output of the dimension $240 \times 240 \times 2$.

Evaluation Metrics for the Sensitivity QoIs

The accuracy of PARC-predicted the average hotspot temperature, total hotspot area, and their rates of change over time is evaluated via several metrics, including root mean squared error (RMSE), Pearson's correlation coefficient (PCC) (42), and Kullback–Leibler divergence (KLD) (43):

$$\text{RMSE}(x, \hat{x}) := \sqrt{\frac{1}{N \times P} \sum_{l=0}^{N} \sum_{k=0}^{P} \left[x_l\left(t_k\right) - \hat{x}_l\left(t_k\right)\right]^2}, \tag{S.1}$$

$$\text{PCC}(x, \hat{x}) := \frac{1}{P} \sum_{k=0}^{P} \frac{1}{\sigma(t_k)\hat{\sigma}(t_k)} \sum_{l=0}^{N} \left[x_l\left(t_k\right) - \mu\left(t_k\right)\right]\left[\hat{x}_l\left(t_k\right) - \hat{\mu}\left(t_k\right)\right], \tag{S.2}$$

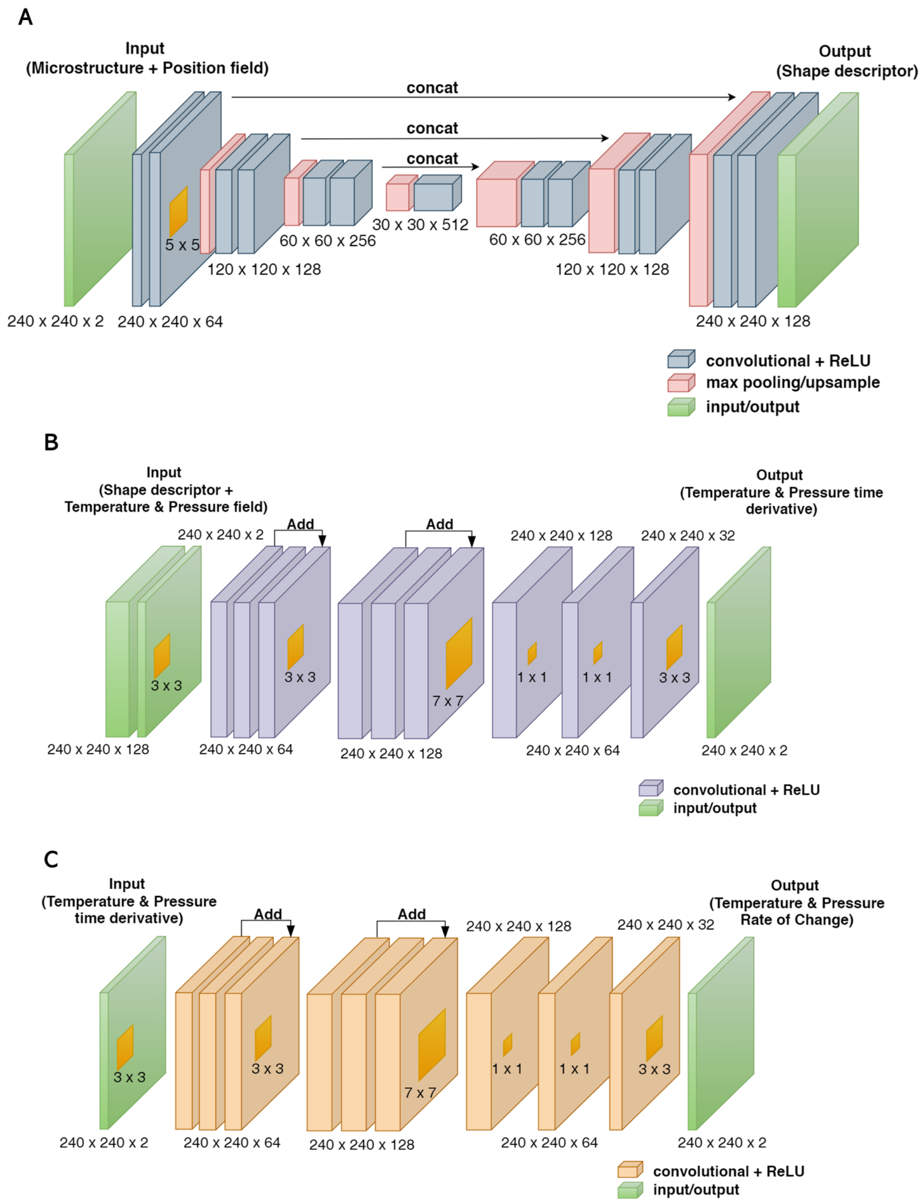

Figure S.1: Architecture design of PARC. (A) The U-Net shape descriptor extractor network. **(B)** Differentiator network. **(C)** Integrator network.

$$\text{KLD}(x, \hat{x}) := \frac{1}{P} \sum_{k=0}^{P} \left[\log \frac{\sigma(t_k)}{\hat{\sigma}(t_k)} + \frac{\hat{\sigma}(t_k) - \{\hat{\mu}(t_k) - \mu(t_k)\}^2}{2\sigma^2(t_k)} - \frac{1}{2} \right]. \tag{S.3}$$

Here, x and $\hat{x}$ denote a sensitivity QoI derived from PARC and DNS, respectively, and μ and σ represent the mean and the standard deviation of the sensitivity QoI, with the similar use of the hat to distinguish PARC and DNS derived values. P is the number of total time steps and N is the number of testing samples.

Saliency Map

Saliency maps (50), or pixel attribution methods, are a way of visualizing CNNs by highlighting image areas that played important roles in the prediction of the outputs. In this paper, we first compute the derivative of the predicted temperature fields *w.r.t.* the individual pixel values of the input microstructure image, to measure the attributions of

microstructural elements to the prediction of the temperature field:

$$\mathbf{G}\left(t_k\right) = \frac{d\mathbf{T}\left(t_k\right)}{d\mathbf{I}}. \tag{S.4}$$

We then compute the sum of such derivatives over all time steps, to account for the time-evolving temperature values:

$$\mathbf{G} = \sum_{k=1}^{P} \mathbf{G}(t_k). \tag{S.5}$$

The raw saliency map produced in this manner can be fuzzy and difficult to interpret. Hence, we apply a threshold value ε to filter out the areas with small or negative gradient values during the backpropagation. The threshold value is selected empirically.

$$\begin{cases} G_{ij} = G_{ij} \quad if \quad G_{ij} \geq \varepsilon, \\ G_{ij} = 0 \quad otherwise. \end{cases} \tag{S.6}$$

Hotspot Ignition and Growth Simulations

The shock-induced energy localization in EM occurs via several mechanisms, including void collapse, plastic dissipation, and intergranular or interface friction. However, under the high shock strength, void collapse is the major source of energy localization while plastic dissipation and inter-granular/interface friction play a minor role in the formation of hotspots. Therefore, the present work focuses on void-collapse-induced hotspot formations.

Mesoscale simulations are performed to simulate the collapse of voids in the microstructure due to the passage of a shock wave. SCIMITAR3D, an in-house multi-material flow solver is used for all simulations. It is a well-tested and validated solver for computing the reactive shock dynamics of energetic materials (18-20) and employs a Cartesian grid-based sharp-interface framework for compressible multi-material flows (57). The governing equations for the motion and deformation of the EM are cast in an Eulerian form (58). The numerical framework has been described in detail in previous works (52-54, 57) and validated against experiments (53) and molecular dynamics simulations (17) for high-speed multi-material shock and impact problems. Details of the numerical treatment can be obtained from the above-cited previous works. Notably, in this work, we study a neat-pressed energetic material which only contains solid HMX phase and void phases in the materials.

To solve the reactive shock dynamics of the pressed HMX system in the present work, the CT images of pressed HMX microstructures are input to the simulation using image processing approaches described in previous work (59). The interfaces between the crystal and void are embedded in the Cartesian grid and tracked using the level-set method and the ghost fluid method (GFM) is used to impose appropriate boundary conditions at the material-material or material-void interfaces. An adaptive local mesh refinement scheme is employed to adequately resolve shocks, reaction fronts and interfaces.The energetic material HMX is modeled as a reactive material employing a 3-equation reduced-order reaction model (9). Note that the material models for HMX are the most up-to-date ones currently available in the literature (17). Among the available reaction chemistry models for HMX, the Tarver 3-equation model has been shown to most closely reproduce experimental data on criticality curves for neat pressed HMX microstructures in recent work (35).

Reactive calculations are performed using methods discussed extensively in previous works (18). The temperature, pressure, and species field data output from the direct numerical simulations are utilized to quantify the response of the pressed material to the imposed shock. In the present context, the quantities of interest (QoIs) for the effect of microstructure on the sensitivity of the material are the evolution of the temperature field and the reaction product mass fraction in the sample. The temperature field $T\left(\mathbf{x}, t\right)$ in the domain measures the intensity of a hot spot resulting from the process of void collapse. Higher temperature hot spots formed due to the collapse of voids in the material lead to high localized energy release rates. The reaction product mass fraction of HMX, *i.e.,* the total mass of solid HMX material converted to gaseous species at any time t, is used to quantify the physio-chemical response of the material and is given by the following equation:

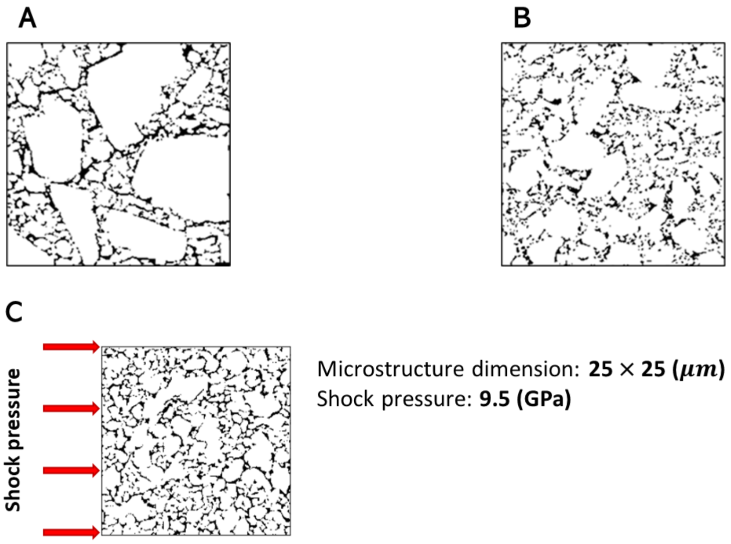

Figure S.2: HMX microstructures and the loading condition of the shock-initiated simulation. (A) Class V microstructure. **(B)** FEM microstructure. **(C)** The shock-initiated simulation is conducted with a shock pressure of 9.5 GPa entering the EM microstructure with the dimension of $25 \times 25 \ (\mu m)$ from the left.

$$F(t) = \frac{M_{reacted}(t)}{M_{HMX}} \tag{S.7}$$

In Eq. S.7, M_{HMX} is the mass of HMX in the total sample prior to the beginning of the chemical reaction process in the material, while $M_{reacted}$ is the mass of complete reaction products resulting from the burning of solid HMX to result in final gaseous reaction products. Complete conversion of the solid HMX to product gases is reached when $F = 1$. The reaction zone defines the hotspot in the domain, which is defined as the region where the temperature of the material exceeds the value of the temperature (T_{bulk}) reached after the passage of a planar shock wave. The hot spot area A_{hs} is an important QoI for determining sensitivity and is calculated as the area of the domain where the temperature $T(\mathbf{x}, t) > T_{bulk}$. The hot spot area is recorded throughout the simulation to track the evolution of the hot spots.

Post-processing of Simulation Data

PARC uses a convolutional neural network (CNN) to associate the original microstructure (a field of voids) with the reactive response (hotspot ignition, growth, and coalescence). However, the original DNS-generated temperature and pressure fields also include the deformation of the microstructure caused by the application of shock load (Fig. S.3A) which makes the training of PARC more difficult. As illustrated in Fig. S.3A, as the shock passes through the domain, the voids are deformed, advected with the local particle velocity and collapse, eventually disappearing and being replaced by hotspots. The hotspots in turn are advected and deformed, assume complex shapes (21, 34), and grow to fuse with neighboring hotspots. Such deformation effects in the state variables and the microstructure will cause difficulties for the prediction of PARC as the computational formulation of the model relies on regression on a fixed grid. For instance, if the hotspot is deformed to an extent greater than the size of CNN receptive field, it is nearly impossible for the CNN to predict the consequent QoI fields in this deformed setting. To overcome the potential difficulty caused by the deformation effect of the DNS-generated fields, we post-process simulation data to transform the original deformed DNS fields back into the non-deformed domain and microstructure. This post-processing of DNS data or 'back-tracking' (Fig. S.3B) utilizes the DNS-computed velocity field associated with each snapshot of the simulations to calculate the microstructural deformation caused

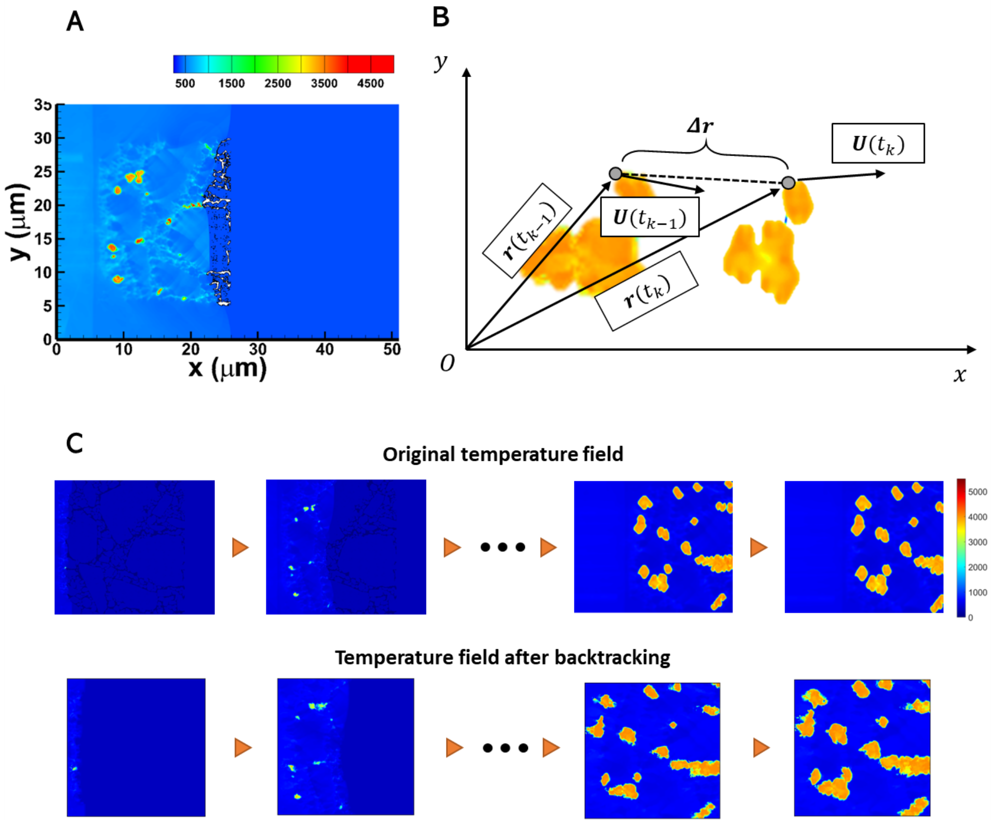

Figure S.3: Backtracking applied to temperature fields. **(A)** The original DNS temperature fields include the deformation of microstructure, causing difficulty in PARC training. **(B)** The back-tracking algorithm using computed velocity field. **(C)** Comparison between original and temperature field after backtracking.

by applied shock. We recursively transform the QoI field in the deformed setting to the non-deformed one by calculating the displacement of every grid point in the domain from its position at the previous snapshot in the sequence of movie frames.

The backtracking of any field values from time step t_k to a previous time step t_{k-1} is conducted under the assumption that each material point lying at a grid node moves with (a known, DNS computed) velocity $\mathbf{u}$ associated with that grid node over the time interval $[t_{k-1}, t_k]$. The displacement $\Delta\mathbf{r}_k$ of a specific grid node associated with the material point at t_k can be computed as

$$\Delta\mathbf{r}_k = 0.5\,(\mathbf{u}_{k-1} + \mathbf{u}_k)\,\Delta t \tag{S.8}$$

where Δt is the time interval between two video frames. Therefore, the position of the grid node, $\mathbf{r}_{k-1}$, at the previous time step t_{k-1} can be computed from its position $\mathbf{r}_k$ at current time step t_k as

$$\mathbf{r}_{k-1} = \mathbf{r}_k - \Delta\mathbf{r}_k \tag{S.9}$$

The computed current DNS-computed field is then collocated with the remapped 'non-deformed' one at that time step. Since the purpose of the backtracking process is to associate the DNS-computed field at a given time t_k to the given initial microstructure at time t_0, the backtracking process is performed recursively from time t_k to time t_0 as described below. Starting from the DNS-computed field at the discrete snapshot time t_k, follow the algorithm:

- **Step 1**: Compute the displacement for every moving grid point of current time step k using Eq. S.8.
- **Step 2**: Compute the position map of all moving grid points at the previous time step k-1 using Eq. S.9

- **Step 3**: Transform the current QoI field to the undeformed configuration by assigning the current value to the remapped location provided in Eq. S.9 at the previous time step $k-1$
- **Step 4**: decrement time level counter k
- **Step 5**: If $k=0$, stop the process; if not, return to Step 1.

The result of the backtracking process is that the spatiotemporally evolving field of voids and hotspots is mapped back to the original fixed Cartesian grid so that the microstructure effectively evolves into a hotspot field in a domain that is undeformed (Fig. S.3C).

Additional Results

Additional results for PARC-predicted temperature and pressure field evolutions are given in Figs. S.4 to S.11. Furthermore, additional results for the extended simulation time experiments are also given in Fig. S.12 and S.13. Finally, additional comparisons with the physics-naïve benchmarks are given in Figs. S.14 and S.15.

Movie clips

Besides the results presented in the figures, we also include movie clips describing the temperature and pressure field evolution predicted by PARC in comparison with ground truth DNS (Movies S.1 to S.9). Addtionally, temperature field evolutions before and after the backtracking procedure is given in Movie S.10.

Movie S.1 Temperature and pressure field evolution movie for test microstructure #1

Movie S.2 Temperature and pressure field evolution movie for test microstructure #2

Movie S.3 Temperature and pressure field evolution movie for test microstructure #3

Movie S.4 Temperature and pressure field evolution movie for test microstructure #4

Movie S.5 Temperature and pressure field evolution movie for test microstructure #5

Movie S.6 Temperature and pressure field evolution movie for test microstructure #6

Movie S.7 Temperature and pressure field evolution movie for test microstructure #7

Movie S.8 Temperature and pressure field evolution movie for test microstructure #8

Movie S.9 Temperature and pressure field evolution movie for test microstructure #9

Movie S.10 Temperature field before and after the backtracking procedure

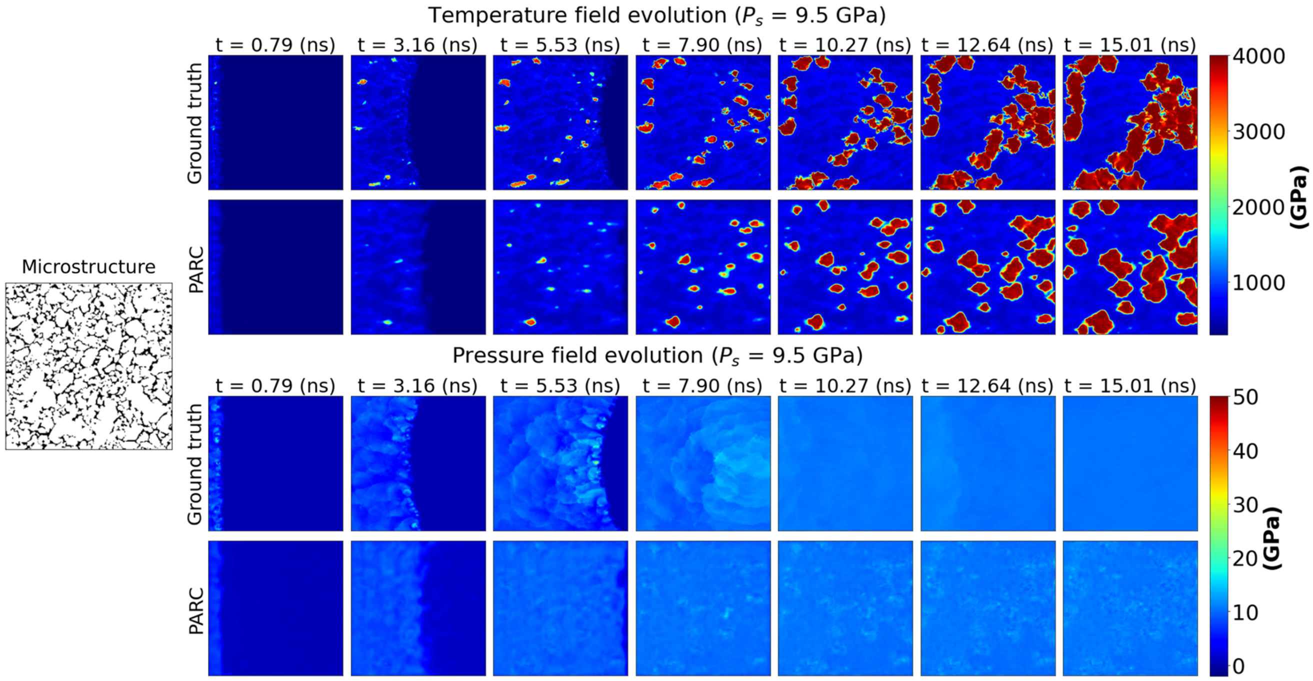

Figure S.4: Temperature and pressure field predictions for test microstructure #2

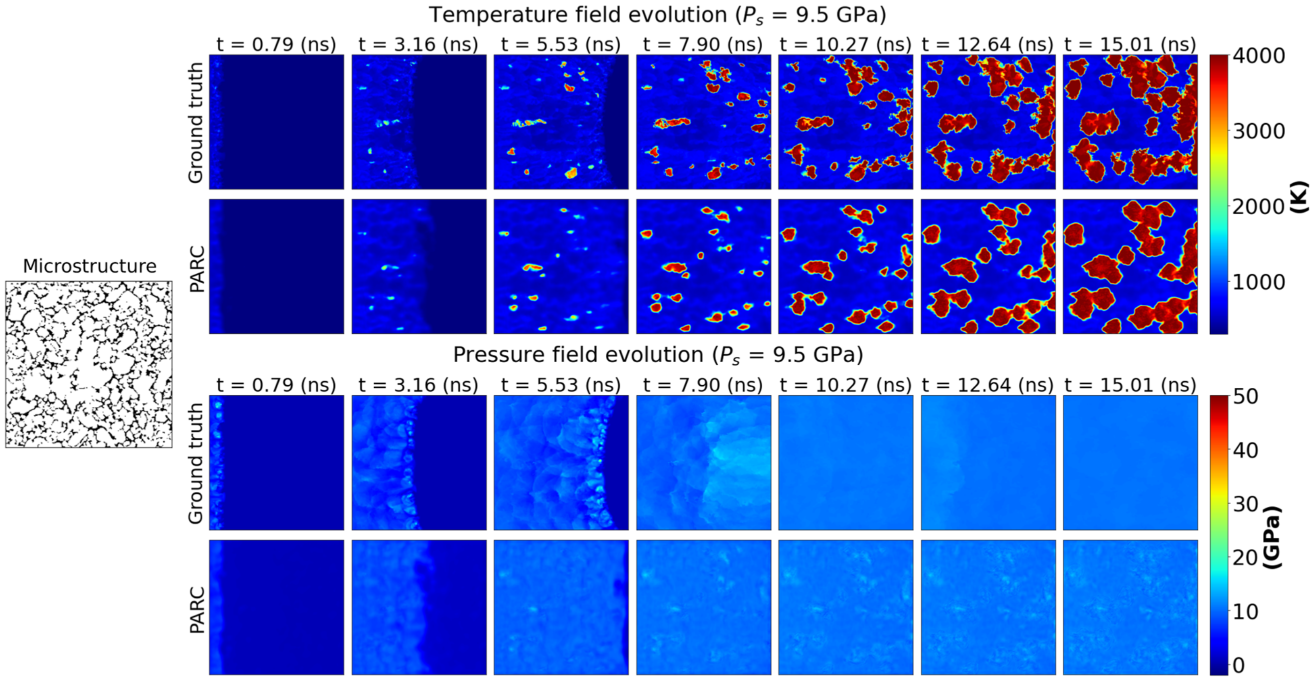

Figure S.5: Temperature and pressure field predictions for test microstructure #3

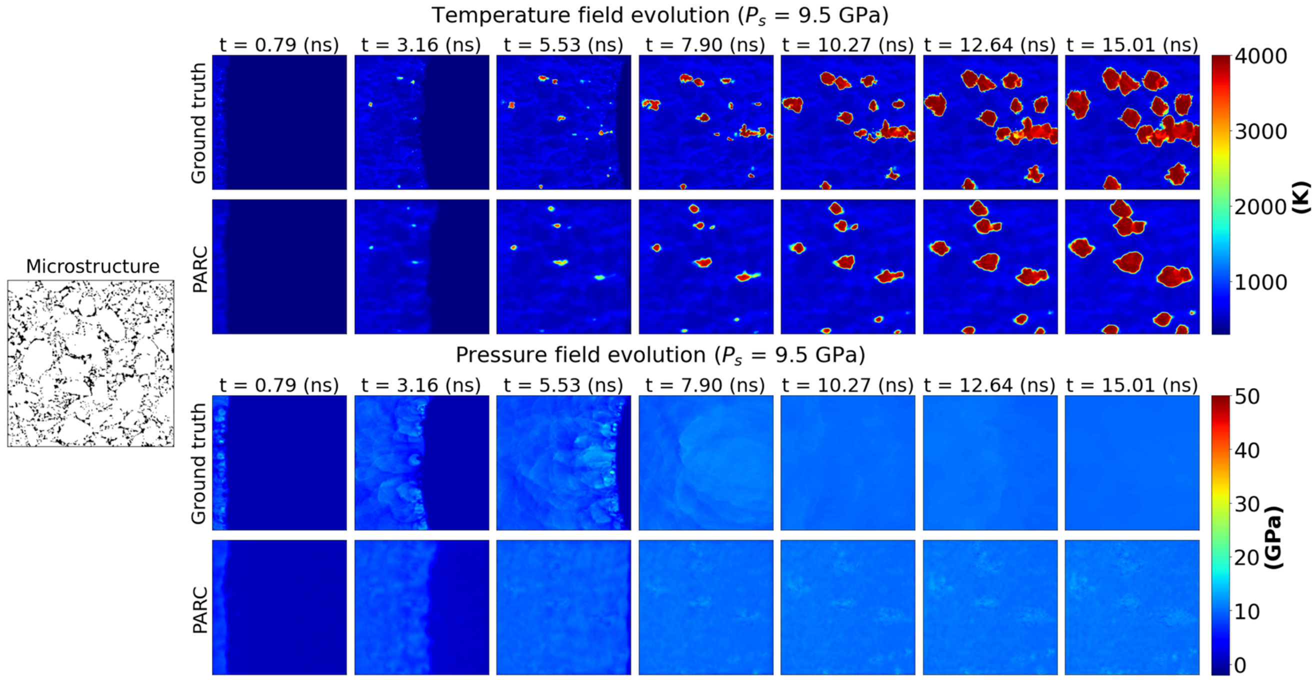

Figure S.6: Temperature and pressure field predictions for test microstructure #4

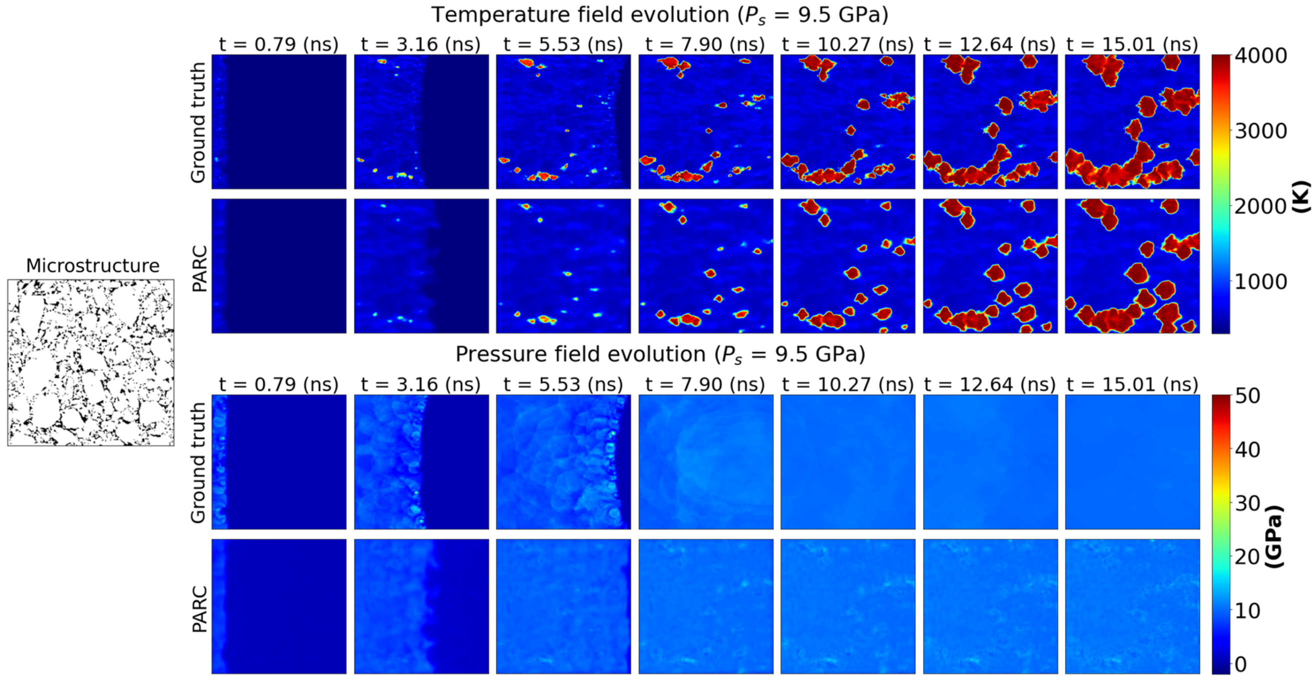

Figure S.7: Temperature and pressure field predictions for test microstructure #5

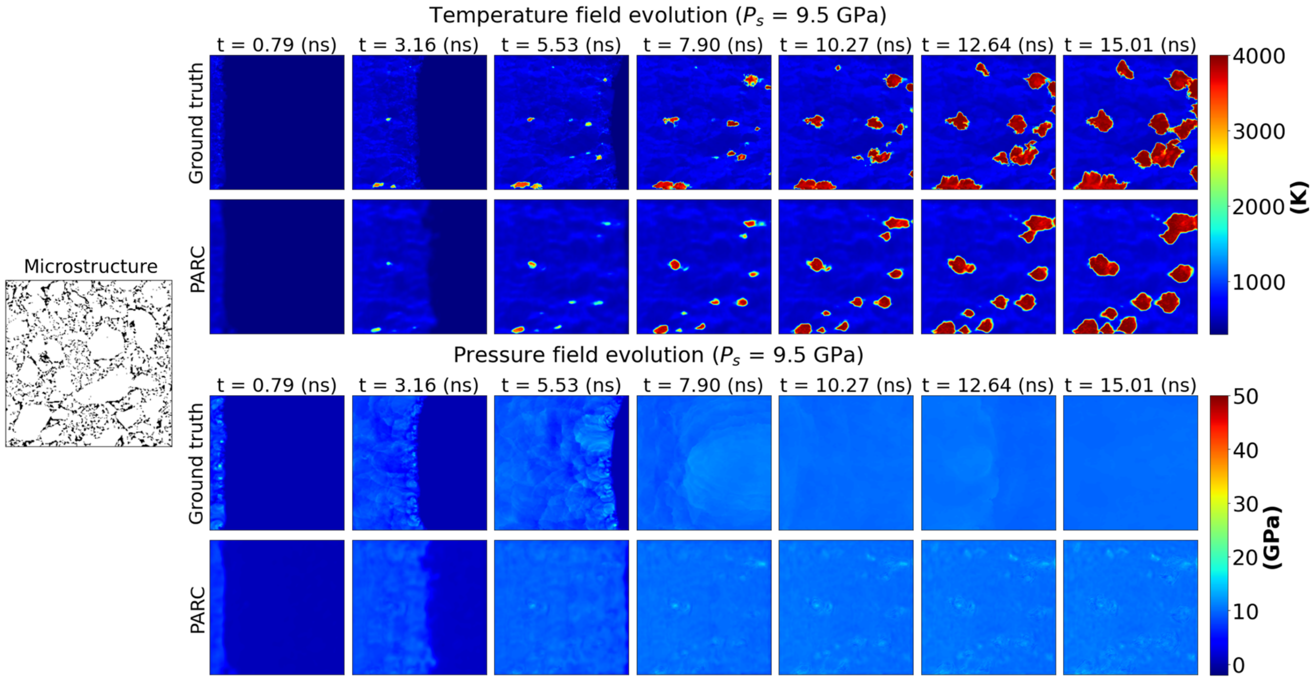

Figure S.8: Temperature and pressure field predictions for test microstructure #6

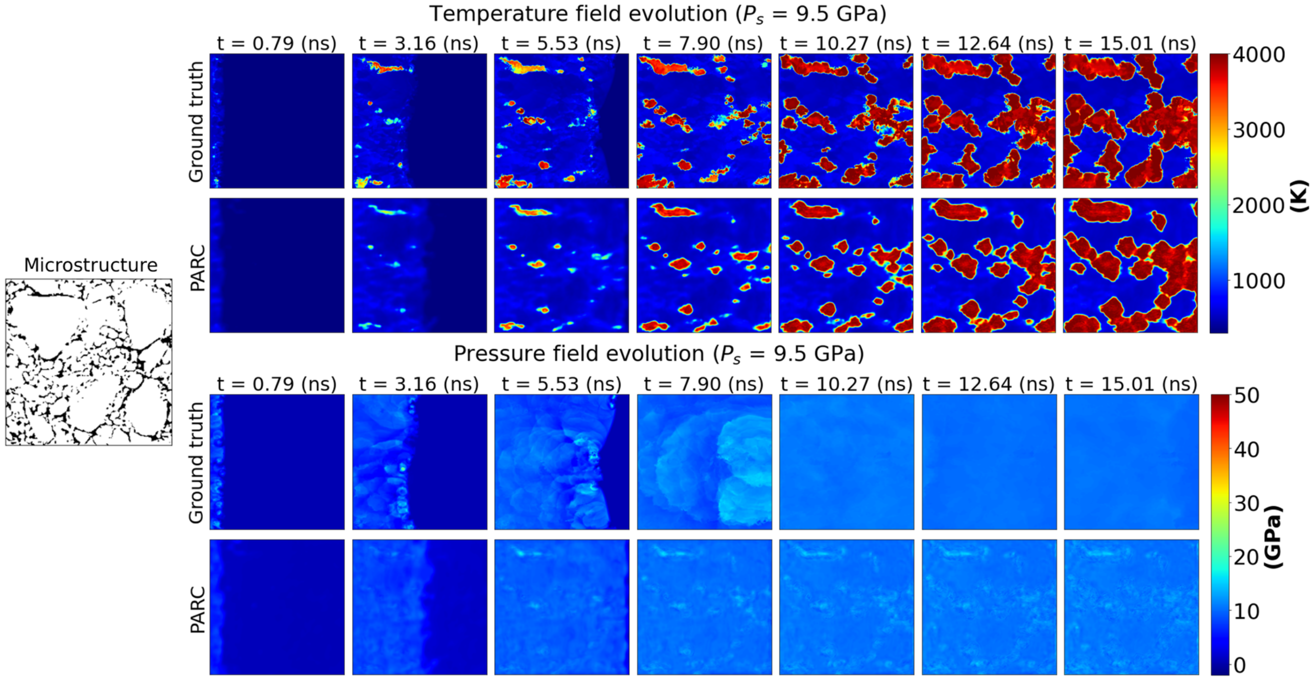

Figure S.9: Temperature and pressure field predictions for test microstructure #7

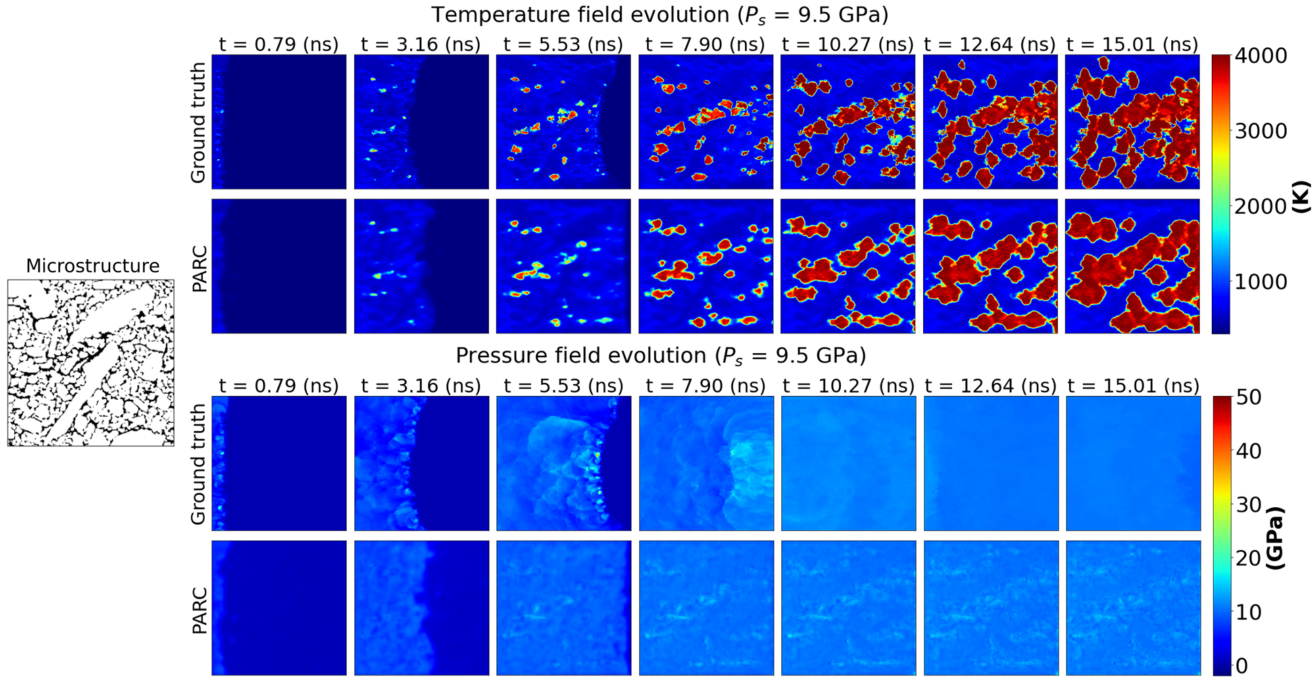

Figure S.10: Temperature and pressure field predictions for test microstructure #8

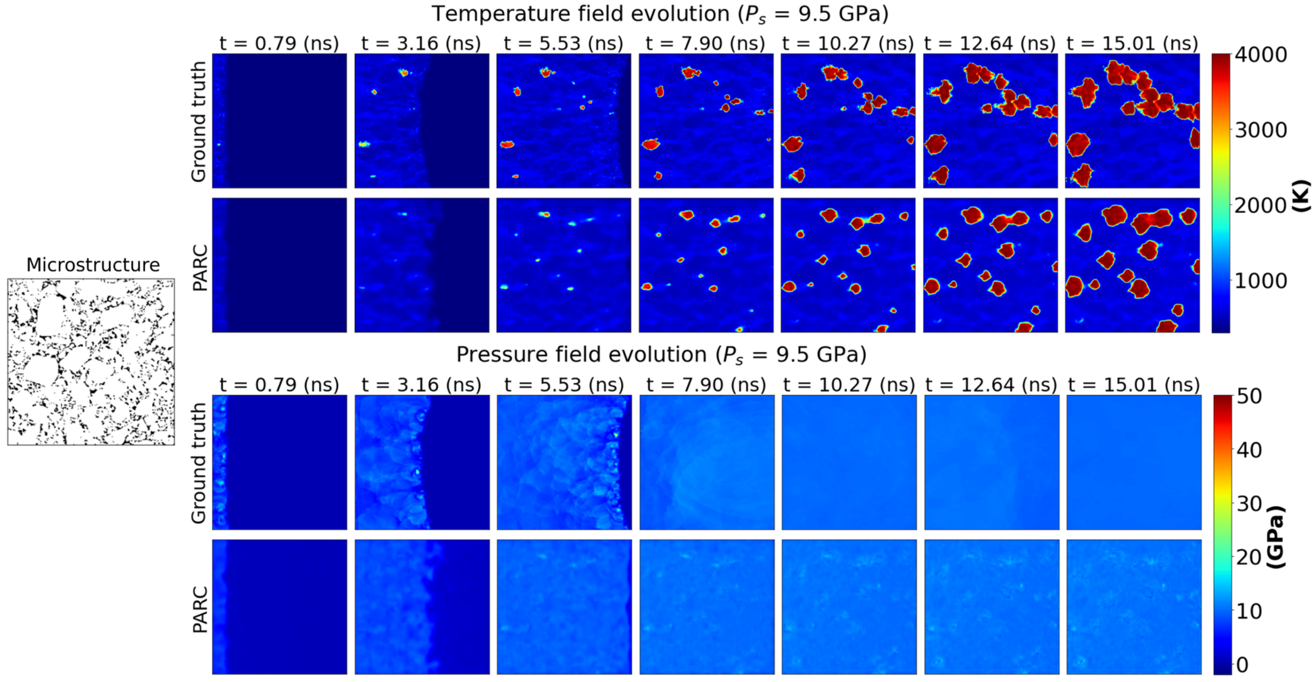

Figure S.11: Temperature and pressure field predictions for test microstructure #9

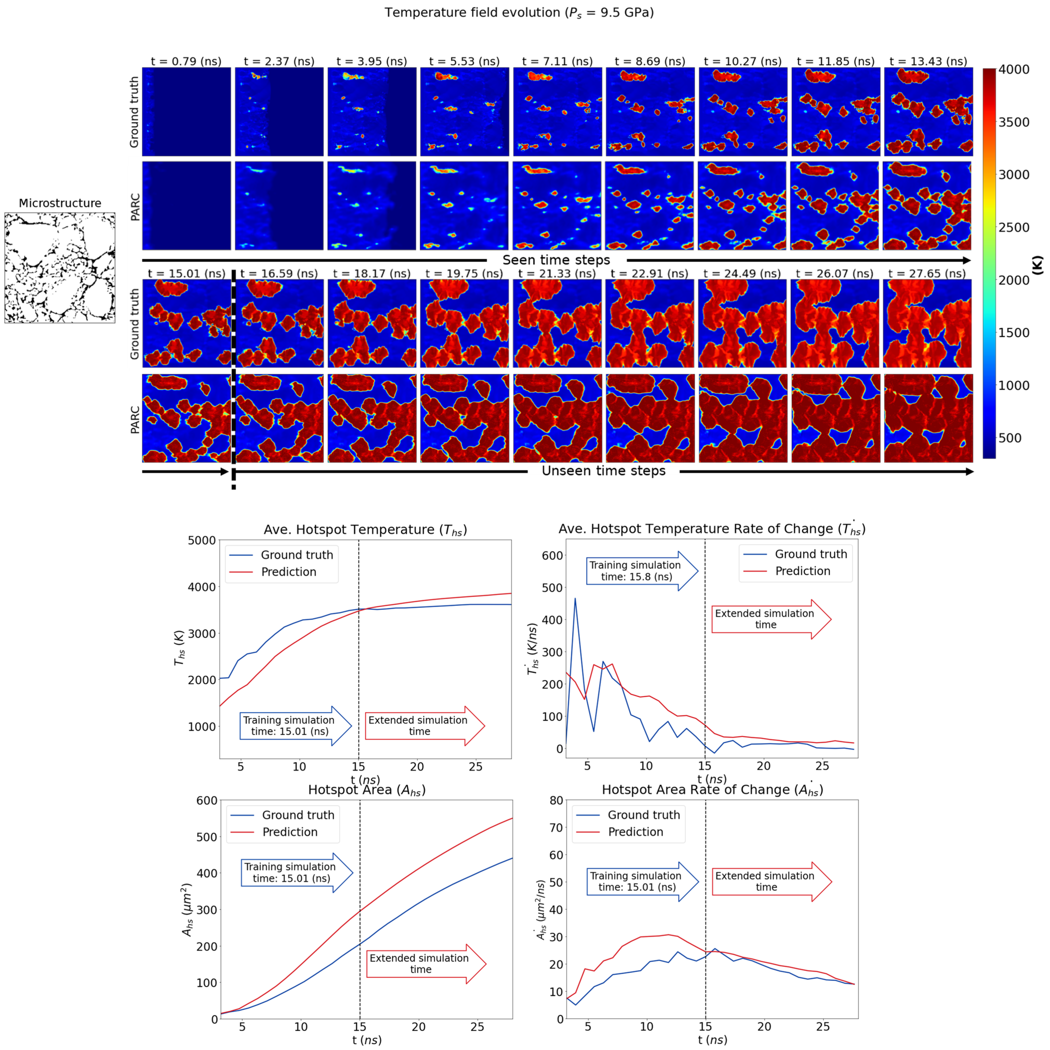

Figure S.12: PARC predictions in unseen time steps for EM microstructure sample #2

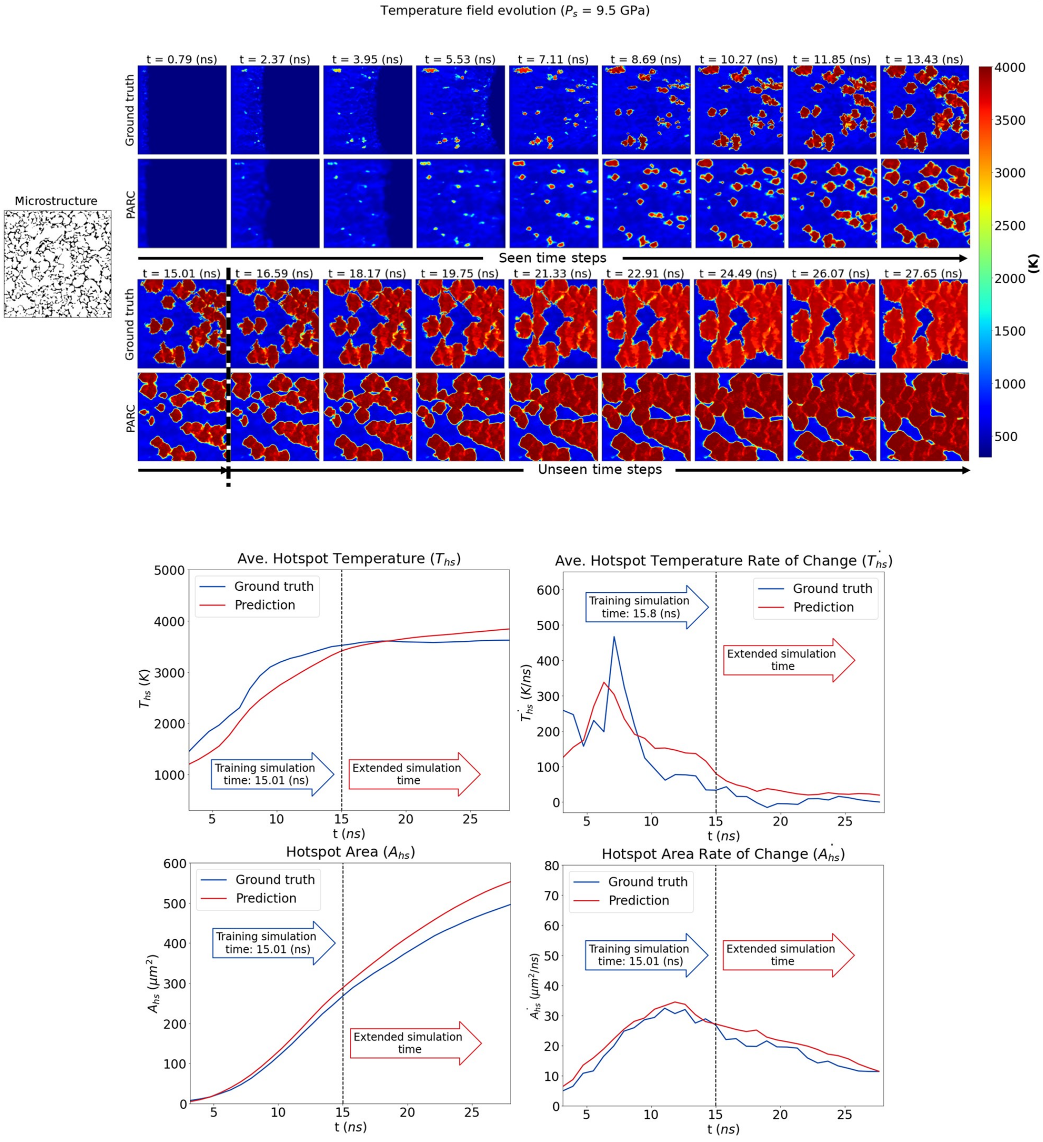

Figure S.13: PARC predictions in unseen time steps for EM microstructure sample #3

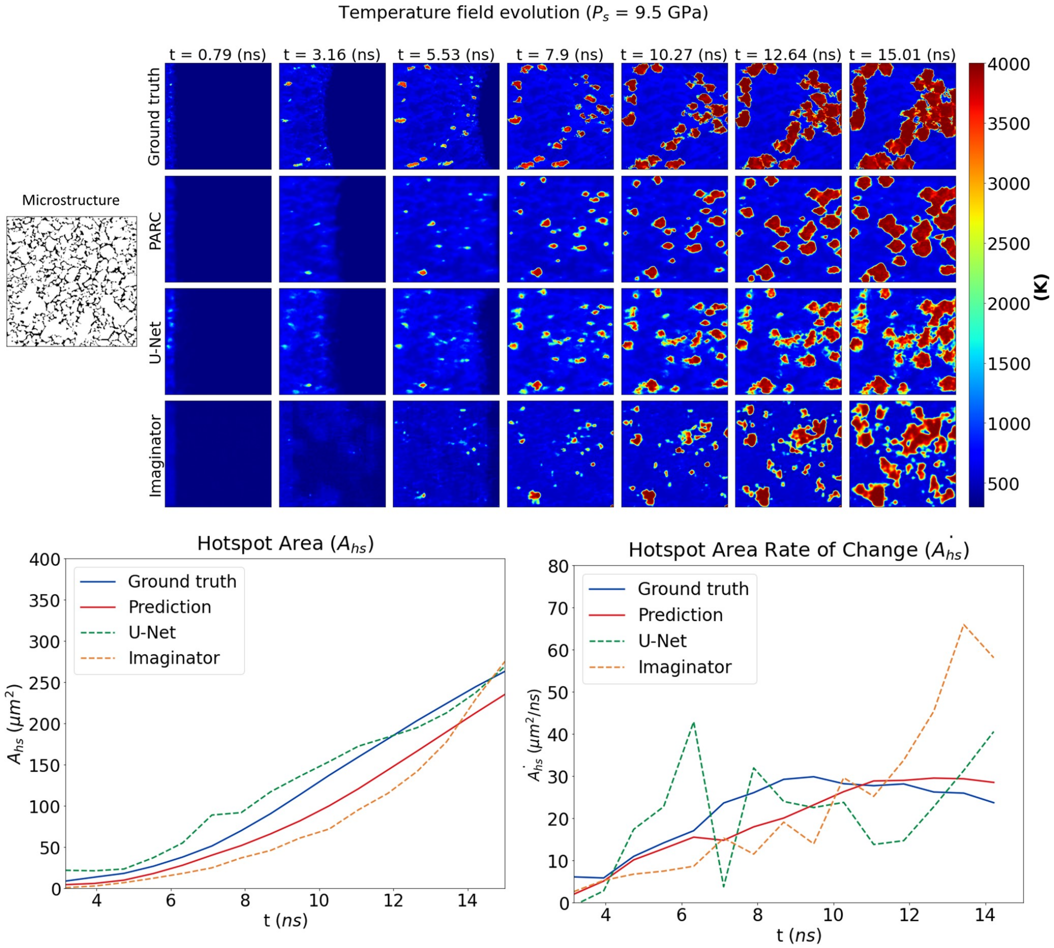

Figure S.14: The benchmarking between PARC and other physics-naïve ML models in hotspot evolution predictions for EM microstructure sample #2

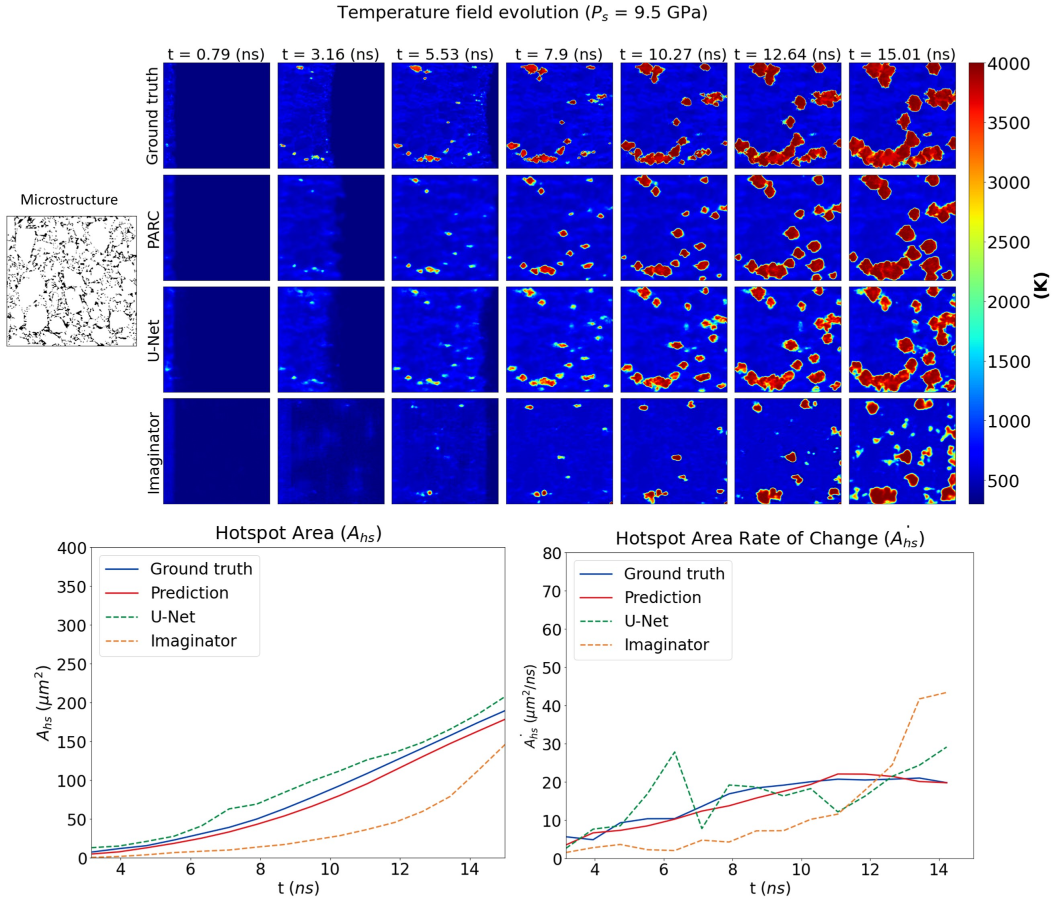

Figure S.15: The benchmarking between PARC and other physics-naïve ML models in hotspot evolution predictions for EM microstructure sample #3

REFERENCES AND NOTES

1. M. R. Baer, Modeling heterogeneous energetic materials at the mesoscale. *Thermochim. Acta* **384**, 351–367 (2002).

2. W. L. Perry, B. Clements, X. Ma, J. T. Mang, Relating microstructure, temperature, and chemistry to explosive ignition and shock sensitivity. *Combust. Flame* **190**, 171–176 (2018).

3. J. T. Mang, R. P. Hjelm, Preferred void orientation in uniaxially pressed PBX 9502. *Propellants Explos. Pyrotech.* **46**, 67–77 (2021).

4. X. Mi, L. Michael, N. Nikiforakis, A. J. Higgins, Effect of spatial distribution of mesoscale heterogeneities on the shock-to-detonation transition in liquid nitromethane. *Combust. Flame* **222**, 392–410 (2020).

5. J. N. Johnson, P. K. Tang, C. A. Forest, Shock-wave initiation of heterogeneous reactive solids. *J. Appl. Phys.* **57**, 4323–4334 (1985).

6. R. B. Frey, The initiation of explosive charges by rapid shear, in *Proceedings of the Seventh Symposium on Detonation* (1982).

7. J. Massoni, R. Saurel, G. Baudin, G. Demol, A mechanistic model for shock initiation of solid explosives. *Phys. Fluids* **11**, 710–736 (1999).

8. R. Menikoff, Pore collapse and hot spots in HMX. *AIP Conf. Proc.* **706**, 393 (2004).

9. C. M. Tarver, S. K. Chidester, A. L. Nichols, Critical conditions for impact- and shock-induced hot spots in solid explosives. *J. Phys. Chem.* **100**, 5794–5799 (1996).

10. J. E. Field, N. K. Bourne, S. J. P. Palmer, S. M. Walley, Hot-spot ignition mechanisms for explosives and propellants. *Philos. Trans. R. Soc. Lond. Ser. A* **339**, 269–283 (1992).

11. S. Chun, S. Roy, Y. T. Nguyen, J. B. Choi, H. S. Udaykumar, S. S. Baek, Deep learning for synthetic microstructure generation in a materials-by-design framework for heterogeneous energetic materials. *Sci. Rep.* **10**, 13307 (2020).

12. H. A. Bruck, R. Gilat, J. Aboudi, A. L. Gershon, A new approach for optimizing the mechanical behavior of porous microstructures for porous materials by design. *Model. Simul. Mater. Sci. Eng.* **15**, 653–674 (2007).

13. K. Alberi, M. B. Nardelli, A. Zakutayev, L. Mitas, S. Curtarolo, A. Jain, M. Fornari, N. Marzari, I. Takeuchi, M. L. Green, M. Kanatzidis, M. F. Toney, S. Butenko, B. Meredig, S. Lany, U. Kattner, A. Davydov, E. S. Toberer, V. Stevanovic, A. Walsh, N.-G. Park, A. Aspuru-Guzik, D. P. Tabor, J. Nelson, J. Murphy, A. Setlur, J. Gregoire, H. Li, R. Xiao, A. Ludwig, L. W. Martin, A. M. Rappe, S.-H. Wei, J. Perkins, The 2019 materials by design roadmap. *J. Phys. D Appl. Phys.* **52**, 13001 (2018).

14. Z. Niu, V. J. Pinfield, B. Wu, H. Wang, K. Jiao, D. Y. C. Leung, J. Xuan, Towards the digitalisation of porous energy materials: Evolution of digital approaches for microstructural design. *Energ. Environ. Sci.* **14**, 2549–2576 (2021).

15. B. L. Holian, T. C. Germann, J.-B. Maillet, C. T. White, Atomistic mechanism for hot spot initiation. *Phys. Rev. Lett.* **89**, 285501 (2002).

16. C. A. Duarte, C. Li, B. W. Hamilton, A. Strachan, M. Koslowski, Continuum and molecular dynamics simulations of pore collapse in shocked β-tetramethylene tetranitramine (β-HMX) single crystals. *J. Appl. Phys.* **129**, 015904 (2021).

17. P. Das, P. Zhao, D. Perera, T. Sewell, H. S. Udaykumar, Molecular dynamics-guided material model for the simulation of shock-induced pore collapse in β-octahydro-1,3,5,7-tetranitro-1,3,5,7-tetrazocine (β-HMX). *J. Appl. Phys.* **130**, 085901 (2021).

18. N. K. Rai, H. S. Udaykumar, Mesoscale simulation of reactive pressed energetic materials under shock loading. *J. Appl. Phys.* **118**, 245905 (2015).

19. N. K. Rai, M. J. Schmidt, H. S. Udaykumar, High-resolution simulations of cylindrical void collapse in energetic materials: Effect of primary and secondary collapse on initiation thresholds. *Phys. Rev. Fluids* **2**, 043202 (2017).

20. N. K. Rai, H. S. Udaykumar, Three-dimensional simulations of void collapse in energetic materials. *Phys. Rev. Fluids* **3**, 33201 (2018).

21. O. Sen, N. K. Rai, A. S. Diggs, D. B. Hardin, H. S. Udaykumar, Multi-scale shock-to-detonation simulation of pressed energetic material: A meso-informed ignition and growth model. *J. Appl. Phys.* **124**, 085110 (2018).

22. N. R. Cummock, J. R. Lawrence, M. Örnek, S. F. Son, The influence of microstructure and conformational polymorph on the drop-weight impact sensitivity of δ-phase HMX. *J. Energ. Mater.* **40**, 445–470 (2021).

23. Y. Liu, J. Xu, S. Huang, S. Li, Z. Wang, J. Li, J. Jia, H. Huang, Microstructure and performance of octahydro-1,3,5,7-tetranitro-1,3,5,7-tetrazocine (HMX) crystal clusters obtained by the solvation-desolvation process. *J. Energ. Mater.* **37**, 282–292 (2019).

24. S. Roy, Y. T. Nguyen, C. Neal, S. Baek, H. S. Udaykumar, Meso-scale simulation of energetic materials. I. A method for generating synthetic microstructures using deep feature representations. *J. Appl. Phys.* **131**, 055904 (2022).

25. P. C. Nguyen, N. N. Vlassis, B. Bahmani, W. Sun, H. Udaykumar, S. Baek, Synthesizing controlled microstructures of porous media using generative adversarial networks and reinforcement learning. *Sci. Rep.* **12**, 9034 (2022).

26. A. Nassar, N. K. Rai, O. Sen, H. S. Udaykumar, Modeling mesoscale energy localization in shocked HMX, part I: Machine-learned surrogate models for the effects of loading and void sizes. *Shock Waves* **29**, 537–558 (2019).

27. S. Roy, N. K. Rai, O. Sen, D. B. Hardin, A. S. Diggs, H. S. Udaykumar, Modeling mesoscale energy localization in shocked HMX, Part II: Training machine-learned surrogate models for void shape and void-void interaction effects. *Shock Waves* **30**, 349–371 (2020).

28. O. Sen, N. J. Gaul, K. K. Choi, G. Jacobs, H. S. Udaykumar, Evaluation of kriging based surrogate models constructed from mesoscale computations of shock interaction with particles. *J. Comput. Phys.* **336**, 235–260 (2017).

29. O. Sen, N. J. Gaul, K. K. Choi, G. Jacobs, H. S. Udaykumar, Evaluation of multifidelity surrogate modeling techniques to construct closure laws for drag in shock-particle interactions. *J. Comput. Phys.* **371**, 434–451 (2018).

30. P. Das, O. Sen, K. K. Choi, G. Jacobs, H. S. Udaykumar, Strategies for efficient machine learning of surrogate drag models from three-dimensional mesoscale computations of shocked particulate flows. *Int. J. Multiph. Flow* **108**, 51–68 (2018).

31. H. R. James, An extension to the critical energy criterion used to predict shock initiation thresholds. *Propellants Explos. Pyrotech.* **21**, 8–13 (1996).

32. F. E. Walker, R. J. Wasley, A general model for the shock initiation of explosives. *Propellants Explos. Pyrotech.* **1**, 73–80 (1976).

33. E. L. Lee, C. M. Tarver, Phenomenological model of shock initiation in heterogeneous explosives. *Phys. Fluids* **23**, 2362 (1980).

34. Y. Nguyen, P. Seshadri, O. Sen, D. B. Hardin, C. D. Molek, H. S. Udaykumar, Multi-scale modeling of shock initiation of a pressed energetic material I: The effect of void shapes on energy localization. *J. Appl. Phys.* **131**, 055906 (2022).

35. S. Roy, N. K. Rai, O. Sen, H. S. Udaykumar, Structure-property linkage in shocked multi-material flows using a level-set-based Eulerian image-to-computation framework. *Shock Waves* **30**, 443–472 (2020).

36. O. Ronneberger, P. Fischer, T. Brox, U-net: Convolutional networks for biomedical image segmentation, in *Medical Image Computing and Computer-Assisted Intervention - MICCAI 2015* (Springer, 2015), pp. 234–241.

37. L. Lu, P. Jin, G. Pang, Z. Zhang, G. E. Karniadakis, Learning nonlinear operators via DeepONet based on the universal approximation theorem of operators. *Nat. Mach. Intell.* **3**, 218–229 (2021).

38. K. Lenc, A. Vedaldi, Understanding image representations by measuring their equivariance and equivalence, in *2015 IEEE Conference on Computer Vision and Pattern Recognition (CVPR)* (IEEE, 2015), pp. 991–999.

39. S. Baek, Y. He, B. G. Allen, J. M. Buatti, B. J. Smith, L. Tong, Z. Sun, J. Wu, M. Diehn, B. W. Loo, K. A. Plichta, S. N. Seyedin, M. Gannon, K. R. Cabel, Y. Kim, X. Wu, Deep segmentation networks predict survival of non-small cell lung cancer. *Sci. Rep.* **9**, 17286 (2019).

40. K. He, X. Zhang, S. Ren, J. Sun, Deep residual learning for image recognition, in *Proceedings of the IEEE Conference on Computer Vision and Pattern Recognition (CVPR)* (IEEE, 2016), pp. 770–778.

41. C. Dong, C. C. Loy, K. He, X. Tang, Image super-resolution using deep convolutional networks. *IEEE Trans. Pattern Anal. Mach. Intell.* **38**, 295–307 (2016).

42. N. Mukhopadhyay, Correlation Coefficient, in *International Encyclopedia of Statistical Science* (2011), pp. 315–318.

43. J. M. Joyce, Kullback-Leibler Divergence, in *International Encyclopedia of Statistical Science* (2011), pp. 720–722.

44. G. E. Karniadakis, I. G. Kevrekidis, L. Lu, P. Perdikaris, S. Wang, L. Yang, Physics-informed machine learning. *Nat. Rev. Phys. Ther.* **3**, 422–440 (2021).

45. Y. Yang, P. Perdikaris, Conditional deep surrogate models for stochastic, high-dimensional, and multi-fidelity systems. *Comput. Mech.* **64**, 417–434 (2019).

46. M. Raissi, P. Perdikaris, G. E. Karniadakis, Physics-informed neural networks: A deep learning framework for solving forward and inverse problems involving nonlinear partial differential equations. *J. Comput. Phys.* **378**, 686–707 (2019).

47. Z. Long, Y. Lu, B. Dong, PDE-Net 2.0: Learning PDEs from data with a numeric-symbolic hybrid deep network. *J. Comput. Phys.* **399**, 108925 (2019).

48. T. Qin, K. Wu, D. Xiu, Data driven governing equations approximation using deep neural networks. *J. Comput. Phys.* **395**, 620–635 (2019).

49. Y. Wang, P. Bilinski, F. Bremond, A. Dantcheva, *2020 IEEE Winter Conference on Applications of Computer Vision (WACV)* (2020), pp. 1149–1158.

50. K. Simonyan, A. Vedaldi, A. Zisserman, Deep inside convolutional networks: Visualising image classification mode, in *Workshop at International Conference on Learning Representations* (2014).

51. H. J. Seltman, *Experimental Design and Analysis* (Carnegie Mellon University Pittsburgh, 2012).

52. N. K. Rai, M. J. Schmidt, H. S. Udaykumar, Collapse of elongated voids in porous energetic materials: Effects of void orientation and aspect ratio on initiation. *Phys. Rev. Fluids* **2**, 043201 (2017).

53. N. K. Rai, S. P. Koundinyan, O. Sen, I. V. Schweigert, B. F. Henson, H. S. Udaykumar, Evaluation of reaction kinetics models for meso-scale simulations of hotspot initiation and growth in HMX. *Combust. Flame* **219**, 225–241 (2020).

54. P. Das, H. S. Udaykumar, A sharp-interface method for the simulation of shock-induced vaporization of droplets. *J. Comput. Phys.* **405**, 109005 (2020).

55. K. He, X. Zhang, S. Ren, J. Sun, Delving deep into rectifiers: Surpassing human-level performance on ImageNet classification, in *2015 IEEE International Conference on Computer Vision (ICCV)* pp. 1026–1034 (IEEE, 2015).

56. D. P. Kingma, J. Ba, Adam: A method for stochastic optimization. arXiv:1412.6980 [**Preprint**] [cs.LG] (30 Jan 2017).

57. A. Kapahi, S. Sambasivan, H. S. Udaykumar, A three-dimensional sharp interface cartesian grid method for solving high speed multi-material impact, penetration and fragmentation problems. *J. Comput. Phys.* **241**, 308–332 (2013).

58. A. G. Kulikovskii, N. V. Pogorelov, A. Y. Semenov, Mathematical aspects of numerical solution of hyperbolic systems, in *Hyperbolic Problems: Theory, Numerics, Applications* (International Series of Numerical Mathematics, 1999), pp. 589–598.

59. S. I. Dillard, J. A. Mousel, L. Shrestha, M. L. Raghavan, S. C. Vigmostad, From medical images to flow computations without user-generated meshes. *Int. J. Numer. Method. Biomed. Eng.* **30**, 1057–1083 (2014).
